# Temperature spectra of conductance of Ge/Si p-i-n structures with Ge quantum dots

**DOI:** 10.1186/s11671-017-1916-0

**Published:** 2017-02-20

**Authors:** Ihor I. Izhnin, Olena I. Fitsych, Anton A. Pishchagin, Andrei P. Kokhanenko, Alexander V. Voitsekhovskii, Stanislav M. Dzyadukh, Alexander I. Nikiforov

**Affiliations:** 1 Scientific Research Company “Carat”, Stryjska St. 202, Lviv, 79031 Ukraine; 2 National Research Tomsk State University, Lenina Av. 36, Tomsk, Russia 634050; 3 A.V. Rzhanov Institute of Semiconductor Physics SB RAS, Akademika Lavrent`eva Av. 13, Novosibirsk, Russia 630090

**Keywords:** GeSi, Molecular-beam epitaxy, Quantum dot, Admittance spectroscopy, Energy levels of spatial quantization, 73.21.La, 73.40.Lq, 73.63.Kv

## Abstract

This work presents results of investigation of Ge/Si p-i-n structures with Ge quantum dots in the i-region by the method of admittance spectroscopy. The structures contain multiple layers with Ge quantum dots separated by thin 5 nm layers of Si in the intrinsic region. Two peaks are observed on the temperature dependences of conductance of the investigated heterostructures. It is revealed that the second peak is broadened and corresponds to a system of closely lying energy levels.

## Background

In the last few years, creating semiconductor structures with new physical properties has been the primary goal of nanotechnology, which has the aim of expanding the limits of applicability of semiconductor materials. The discovery of new physical properties allows for creating new devices using advanced technology of silicon microelectronics [1–4]. Also, in recent years, the interest in photoelectric properties of Ge/Si heterostructures (primarily designed for the spectral range of 1.3–1.55 μm) has increased. New types of photodetectors based on silicon-germanium low-dimensional heterostructures using intrasubband and intersubband transitions are intensively being developed. Such devices may be used in optoelectronic communication systems and remote monitoring [2, 5]. Large attention to Ge/Si structures is caused due to the possibility of their use in solar energy. Ge/Si heterostructures with Ge quantum dots have turned out to be promising for creating high-efficiency solar cells. Theoretical estimations predict efficiency of 53% for solar cells based on Si with Ge quantum dots [6]. Improving the efficiency of devices based on Ge/Si heterostructures with Ge quantum dots is possible due to the effects of spatial quantization.

Nowadays, admittance spectroscopy is extensively used to obtain the information about some properties of semiconductor heterostructures with quantum dots. A large number of studies have been devoted to investigating structures containing Schottky barriers by the methods of admittance spectroscopy [7–9]. But it should be noted that studies of the characteristics of p-i-n structures based on Ge/Si material system by the method of admittance spectroscopy are still extremely rare. However, for device applications such as solar cells and photodetectors, p-i-n-structures are very important. In this work, we show the possibility of studying p-i-n-structures based on Si with Ge quantum dots by admittance methods.

### Methods

In this paper, we present the experimental results on synthesis of Si/Ge p-i-n structures with Ge quantum dots in the i-region and their investigation by the method of admittance spectroscopy.

The samples were fabricated by molecular beam epitaxy in an ultra-high vacuum installation “Katun-C” in the Institute of Semiconductor Physics. Evaporation of silicon and germanium was carried out by electron beam evaporators, and the dopants (Sb and B) were evaporated from effusion cells. The analytical part of the epitaxy chamber consists of a quadrupole mass spectrometer, a quartz thickness gauge, and a reflection high energy electron diffractometer. The growth of Ge quantum dots was carried out on Si(100) substrates with misorientation less than 0.5° at 500 °C. The growth rates of Ge and Si layers were 0.025 and 0.113 nm/s, respectively.

For investigations, samples of photoelements with different size and topology of the active region were prepared. Heavily doped p- and n-regions were obtained by doping with B and Sb, respectively. The remaining regions including quantum dots were not intentionally doped and served as i-layers. Ohmic contacts to the p- and n-regions were fabricated by evaporation of Al layers followed by thermal annealing at 450 °C.

We have studied 3 types of samples. All of them are p-i-n structures, but type 1 samples are p-i-n structures without layers of quantum dots, while type 2 and type 3 samples contain multiple layers of Ge quantum dots in the intrinsic region. The height of Ge hut-clusters is 1.5–3 nm, lateral size is 10–40 nm, and surface density is ~10^11^ cm^−2^. The intrinsic region of type 2 samples contains 30 layers of 6 monolayer Ge quantum dots separated by 5 nm silicon layers. The intrinsic region of type 3 samples is similar to that of type 2, but every 10 layers of Ge quantum dots are additionally separated by 100 nm of Si.

Measurements were performed on an automated admittance spectroscopy installation [10]. During one cycle of temperature scanning, measurements of the frequency and temperature dependences of capacitance and conductance, as well as of current-voltage characteristics of the studied structures were carried out in the temperature range 10–300 K.

## Results and discussion

By analogy with deep levels in semiconductors, the principle of admittance spectroscopy of structures with quantum dots is based on measuring the complex conductivity of the system: it changes when discrete energy levels recharge due to emission of charge carriers and their capture by localized states. The temperature spectra of conductance (*G-T*) at different frequencies of the test signal and different bias voltages are often considered to be the most informative.

**Fig. 1:**
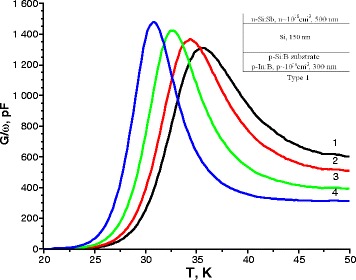
Temperature spectra of conductance for sample type 1 measured at the applied bias voltage of –0.5 V and various frequencies of the test signal *1* 2000 kHz, *2* 1500 kHz, *3* 1000 kHz, and *4* 500 kHz. The *inset* shows the structure of samples

In the temperature dependence of conductance of type 1 samples, a maximum was observed at low temperatures (25–40 K). The observed maximum of conductance corresponds to a discrete energy level. The position of this peak shifts on the temperature scale as the frequency of the applied signal changes (Fig. 1). Conductance peak position for these samples remains constant with changes in the applied bias voltage.

**Fig. 2:**
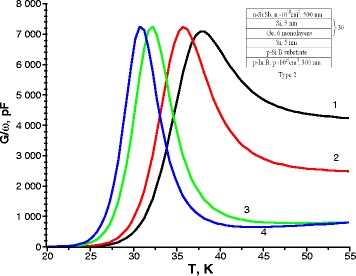
Temperature spectra of conductance for sample type 2 measured at the applied bias voltage of –0.5 V and various frequencies of the test signal *1* 100 kHz, *2* 50 kHz, *3* 10 kHz, and *4* 5 kHz. The *inset* shows the structure of samples

**Fig. 3:**
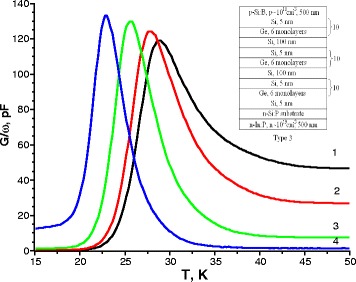
Temperature spectra of conductance for sample type 3 measured at the applied bias voltage of –0.5 V and various frequencies of the test signal *1* 1000 kHz, *2* 500 kHz, *3* 100 kHz, and *4* 10 kHz. The *inset* shows the structure of samples

Similar results were obtained in studies of type 2 and type 3 samples. Figures 2 and 3 show the temperature conductance spectra of type 2 and type 3 samples measured at different voltages and different frequencies.

The charge carrier emission rate from a discrete level is determined by the expression (1):

1 $$ e= A{T}^2 \exp \left(-\frac{E_a}{kT}\right), $$

where *T* is the temperature, *k* is the Boltzmann’s constant, *E*_*a*_ is the activation energy, *A* is the pre-exponential factor which does not depend on the temperature and is proportional to the capture cross section of the charge carriers at the localized state. The contribution of the discrete energy levels to the conductance is determined by the following expression [10]:

2 $$ {G}_T\sim \frac{ e\omega}{e^2+{\omega}^2}{\left(\frac{1}{2{V}_b}\right)}^{\frac{1}{2}}, $$

where *ω* is the angular frequency of the test signal and *V*_*b*_ is the applied voltage.

The main characteristic of a discrete energy level is the charge carrier activation energy. To determine the activation energy, the experimental points are plotted in coordinates *ln e = f(1/T)*, called an Arrhenius plot. In the Arrhenius coordinates, dependence (1) is a straight line.

**Fig. 4:**
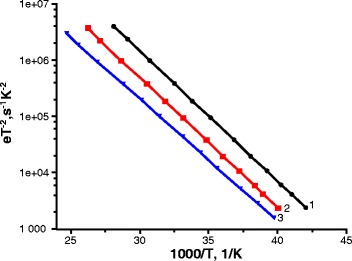
Arrhenius plots built for the positions of the maxima of the temperature spectra of conductance of type 1 samples at various bias voltages *1* −1 V, *2* −0.5 V, and *3* −0 V

The *GT/ω* value has a maximum at *ω = e*. By plotting maxima *T*_max_ in coordinates *ω = f(1/T)*, we determine the activation energy characterizing the position of the energy levels. We have plotted point with coordinates *ln(e/T* ^*2*^ *)*, *1/T*_max_ for each frequency, and have built the approximating straight line. Then, we have calculated activation energy from the slope of the line. Figure 4 shows the Arrhenius plot for temperature spectra of conductance of type 1 samples.

**Fig. 5:**
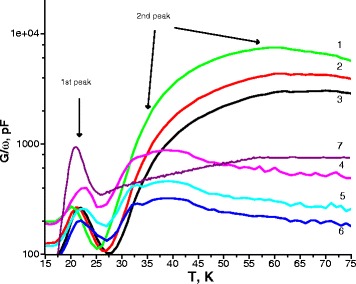
Temperature spectra of conductance of type 3 sample, measured at the voltages of +1 V (*1*–*3*) and +2 V (*4*–*6*) at various frequencies *1* 1 kHz, *2* 2 kHz, *3* 3 kHz, *4* 1 kHz, *5* 2 kHz, *6* 3 kHz. *7* Temperature spectra of conductance of type 1 sample, measured at the applied bias voltage of +2 V and frequency of the test signal of 500 kHz

In a further study of type 3 samples, a conductance peak at low positive bias was also detected (peak 2 at Fig. 5). This maximum is observed only at positive biases at higher temperatures and is most pronounced at low frequencies, while the first maximum is also observed at negative biases.

**Fig. 6:**
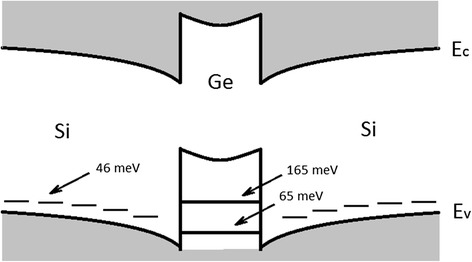
Schematic image of the band structure and energy levels of Ge quantum dots in Si

Processing temperature spectra leads to a typical family of Arrhenius plots suitable for finding activation energies of the emission process. For all samples, activation energies were calculated. For the first peak of conductance calculated activation energies of type 1, type 2 and type 3 samples do not depend on the applied bias voltage and are equal to 38 ± 5, 42 ± 4, and 46 ± 4 meV, respectively. For the second peak, the calculated activation energy at the bias voltage of 1 V is 65 ± 10 meV; at the bias voltage of 2 V, it is 165 ± 30 meV. Figure 6 shows the band structure and energy levels of Ge quantum dots in Si.

We observed first peak at low temperatures (~20–40 K) for all bias voltages. From the presented figures, it is seen that the position of this maximum in the temperature dependence of conductance and corresponding activation energies do not show a pronounced dependence on the applied bias voltage. Recharging of the level occurs with fixed bias voltage *V*_*b*_. The charge carrier emission rate from this level decreases at lower temperatures, so with a decrease in the frequency of the test signal, the condition of maximum conductance is achieved at lower temperatures and at different frequencies.

We assume that the first peak may be associated with the impurity level in Si.

Yakimov et al. [7] conducted research on Si Schottky barrier structure with several layers of Ge quantum dots by admittance spectroscopy method at low temperatures. They revealed the presence of two maxima in the temperature dependence of conductance. The first maximum did not depend on the applied bias and corresponded to a doping level.

We received another evidence of this from the compared temperature spectra for type 2 and type 3 samples with temperature spectra for type 1 samples without Ge quantum dots. The temperature spectra for type 1 samples have the maximum at the same temperatures. So, the discrete energy level with activation energy of ~35–50 meV, corresponding to the first peak of the temperature dependence of conductance, is not related to the spatial quantization in quantum dots.

The second peak can be explained by the presence of spatial quantization levels in the system associated with Ge quantum dots. This peak is broadened and probably corresponds not to a single discrete but to a system of closely lying levels, due to the inhomogeneity of such parameters of quantum dots as their lateral size, height, shape, and density in the array. We explain the appearance and modification of peaks by the following fact. With a change in the applied voltage, the electrochemical potential occasionally crosses the discrete energy levels and it produces oscillations in the charge density distribution. The reason for this is the thermionic emission of charge carriers from a discrete level. Discrete level gives partial charge density increment. This increment of charge leads to an increase in the external circuit current measured as a change in conductance of a sample.

Thus, analysis of the results and literature data allows for making the following assumptions. The lack of size quantization effect in type 2 structure (absence of the second peak in the temperature dependence of conductivity) may be due to the accumulation of strains during the growth of successive Ge layers, which leads to an increase in the size of the quantum dots [11], and/or non-uniform strain relaxation, resulting in a large number of defects [12]. Introduction of thick Si spacer layers in type 3 structure should significantly reduce these factors, which leads to the appearance of the size quantization in Ge quantum dots and, thus, the appearance of the second peak in the temperature dependence of conductivity.

## Conclusions

This paper shows the possibility of studying Si p-i-n-structures with Ge quantum dots by the method of admittance spectroscopy. We have investigated a number of Si p-i-n-structure samples with different type of i-layers. Only for sample containing 30 layers of 6 monolayer Ge quantum dots separated by 5 nm silicon layers in which every 10 layers of Ge quantum dots were additionally separated by 100 nm of Si (type 3) was observed two peaks on the temperature dependences of conductance. The first peak was observed at any bias voltage, although the second peak was observed in the narrow voltage range. Position of the first peak does not depend on the applied bias voltage, the second peak shifts on temperature scale with changing the bias voltage from 1 to 2 V. We have calculated activation energies for both peaks. The first peak of the temperature dependence of conductance may be associated with the impurity level in Si (46 meV). The second peak can be explained by the presence of spatial quantization levels in the system associated with Ge quantum dots 65 and 165 meV. Applying thick Si spacers in type 3 structure allows for eliminating the strain and for obtaining the highest degree of uniformity of the array of quantum dots with the minimum number of defective islands, which determines the appearance of the size quantization in the Ge quantum dots.
